# Impact of implantation depth and calcium burden on infranodal conduction delay after transcatheter aortic valve replacement

**DOI:** 10.1016/j.hroo.2023.12.003

**Published:** 2023-12-18

**Authors:** Andrea Papa, Teodor Serban, Ivo Strebel, Sven Knecht, Corinne Isenegger, Thomas Nestelberger, Christoph Kaiser, Gregor Leibundgut, Philipp Haaf, Beat Schaer, Philipp Krisai, Stefan Osswald, Christian Sticherling, Michael Kühne, Patrick Badertscher

**Affiliations:** Department of Cardiology and Cardiovascular Research Institute Basel, University Hospital Basel, University of Basel, Basel, Switzerland

**Keywords:** HV interval, Implantation depth, Infranodal conduction delay, Left ventricular outflow tract calcification, Transcatheter aortic valve replacement

## Abstract

**Background:**

Infranodal conduction disorders are common after transcatheter aortic valve replacement (TAVR). Risk factors are incompletely understood.

**Objective:**

The purpose of this study was to assess the impact of valve implantation depth and calcium burden of the device landing zone on infranodal conduction intraprocedure pre- and post-TAVR.

**Methods:**

In all patients undergoing TAVR between June 2020 and June 2021, the His-ventricle (HV) interval was measured pre- and post-valve deployment. The difference between the 2 measurements defined delta HV, whereas infranodal conduction delay was defined as HV interval >55 ms. Valve implantation depth was measured as the distance between the aortic annular plane and the ventricular prosthesis end. Calcium burden was quantified as the volume of calcium in 6 regions of interest: the non-, right, and left coronary cusps (NCC, RCC, and LCC, respectively) and the corresponding regions of the left ventricular outflow tract (LVOT) underlying each cusp (LVOT_NCC_, LVOT_RCC_, LVOT_LCC_, respectively).

**Results:**

Of 101 patients (mean age 81 ± 5.7 years; 47% women), 37 demonstrated infranodal conduction delay intraprocedure post-TAVR. Overall, mean implantation depth was 5 ± 3.1 mm, median calcium volume was 2080 mm^3^ [interquartile range 632–2400]. Delta HV showed no correlation with implantation depth or calcium burden (r = –0.08 and r = 0.12, respectively). However, LVOT_NCC_ calcification was a significant predictor for infranodal conduction delay post-valve deployment in a multivariable logistic regression model (odds ratio 1.62 per 100-mm^3^ increase (95% confidence interval 1.06–2.69; *P* = .04).

**Conclusion:**

Assessment of LVOT_NCC_ calcification may identify patients at risk for infranodal conduction delay after TAVR, whereas implantation depth did not predict infranodal conduction delay.

**Key Findings**

▪ Patients with infranodal delay after transcatheter aortic valve replacement (TAVR) show higher calcium volume at the level of the left ventricular outflow tract (LVOT), especially in the region underlying the noncoronary cusp (LVOT_NCC_), in comparison to patients with normal atrioventricular conduction.
▪ LVOT_NCC_ calcification was a significant predictor of infranodal conduction delay post-TAVR.
▪ Calcium volume at the level of the aortic valve did not differ between patients with infranodal delay and those with normal HV interval.
▪ In comparison to patients with normal atrioventricular conduction, implantation depth was similar in patients with a prolonged HV interval, whereas implantation depth was significantly deeper in patients undergoing periprocedural pacemaker implantation.

## Introduction

Transcatheter aortic valve replacement (TAVR) has become an established treatment for elderly patients with severe aortic stenosis irrespective of perioperative risk.^1^ However, because of the proximity of the device landing zone (DLZ) to the cardiac conduction system,^2^ new-onset conduction disturbances requiring permanent pacemaker implantation (PMI) remain among the most frequent complications (6%–28%).

Valve implantation depth is a crucial factor for an optimal outcome after TAVR.^3^ Whereas a high implantation depth is associated with a higher risk of coronary obstruction or valve embolization,^4^ a low implantation depth reportedly is associated with an increased need for PMI.^5^ However, the optimal implantation depth is not yet known and needs to be balanced to avoid these complications.

Aortic valve calcification has also been identified as a predictor of conduction disturbances after TAVR.^6^ However, little is known about how the distribution of calcium within the DLZ directly impacts on atrioventricular (AV) nodal conduction parameters.

Although current guidelines recommend performing electrophysiological (EP) testing for risk stratification of patients with new left bundle branch block (LBBB) 3 days after TAVR,^7^ EP testing pre- and post-valve deployment would enable direct assessment of periprocedural injury to the conduction system.^8^

The purpose of this study was to investigate the impact of implantation depth and calcium burden of the DLZ on infranodal conduction properties during and after TAVR.

## Methods

### Study design and patient population

We conducted a retrospective analysis of prospectively collected data from the Swiss TAVR Registry (SwissTAVI Registry: Prospective, National, Multi-Center Registry of Patients Undergoing Transcatheter Aortic Valve Implantation, ClinicalTrials.gov Identifier: NCT01368250) of consecutive patients undergoing TAVR at our institution from June 2020 to June 2021. For procedural planning, patients underwent transthoracic echocardiography, coronary angiography, and electrocardiography-triggered multislice computed tomographic (CT) scan of the aorta. EP study was conducted during TAVR (pre-valve and post-valve deployment), with an additional EP study performed the day after if LBBB persisted (new-onset and pre-existing LBBB included). Patients who previously had a PMI, had valve-in-valve TAVR, experienced periprocedural death, or had missing EP measurements were excluded from the study. Valve types included self-expandable Evolut R and Evolut R Pro (Medtronic, Dublin, Ireland), Acurate NEO (Boston Scientific, Marlborough, MA), balloon-expandable Sapien 3 and Sapien 3 ultra (Edwards Life Science, Irvine, CA), or mechanically expandable Lotus Edge (Boston Scientific, Marlborough, MA). All patients provided written informed consent, and the local ethics committee approved the study protocol.

### EP measurements

A limited EP study was performed before and after valve deployment in all patients.^8^ Intracardiac measurements were obtained using a portable EP system (Cardiotek EP Tracer 70, Medtronic, Dublin, Ireland) in combination with the quadripolar diagnostic catheter (CRD 5F catheter, St. Jude Medical, Saint Paul, MN) used as a temporary pacemaker (PM) wire during TAVR. After the catheter was withdrawn from the ventricle to the His position, HV interval was measured over 3 consecutive beats using the electronic calipers with a sweep speed of 100 mm/s. This was performed immediately before implantation (pre-valve deployment) and after invasive hemodynamic confirmation of correct valve placement (post-valve deployment) (Supplemental Figure S1). Differences in AV nodal conduction were calculated for AH, HV, and PR interval and QRS duration between the measurements pre- and post-valve deployment as well as the day after TAVR. Infranodal conduction delay was defined as HV interval >55 ms. Patients then were stratified according to HV interval into a group with normal conduction or a group with prolonged HV interval (>55 ms). Delta HV was defined as the intraprocedural difference of HV intervals post- and pre-valve deployment. Patients with and without LBBB post-valve deployment also were stratified into an LBBB and a non-LBBB group, respectively. In patients with persistent LBBB the day after TAVR, EP study was performed in the electrophysiology laboratory (Axiom Sensis EP System, Siemens Healthineers, Erlangen, Germany). Finally, a permanent PM was implanted in patients who developed a complete AV block during procedure. HV interval measurement was not possible in these patients, so they were considered for secondary analysis.

### Measurements of valve implantation depth

**Figure 1:**
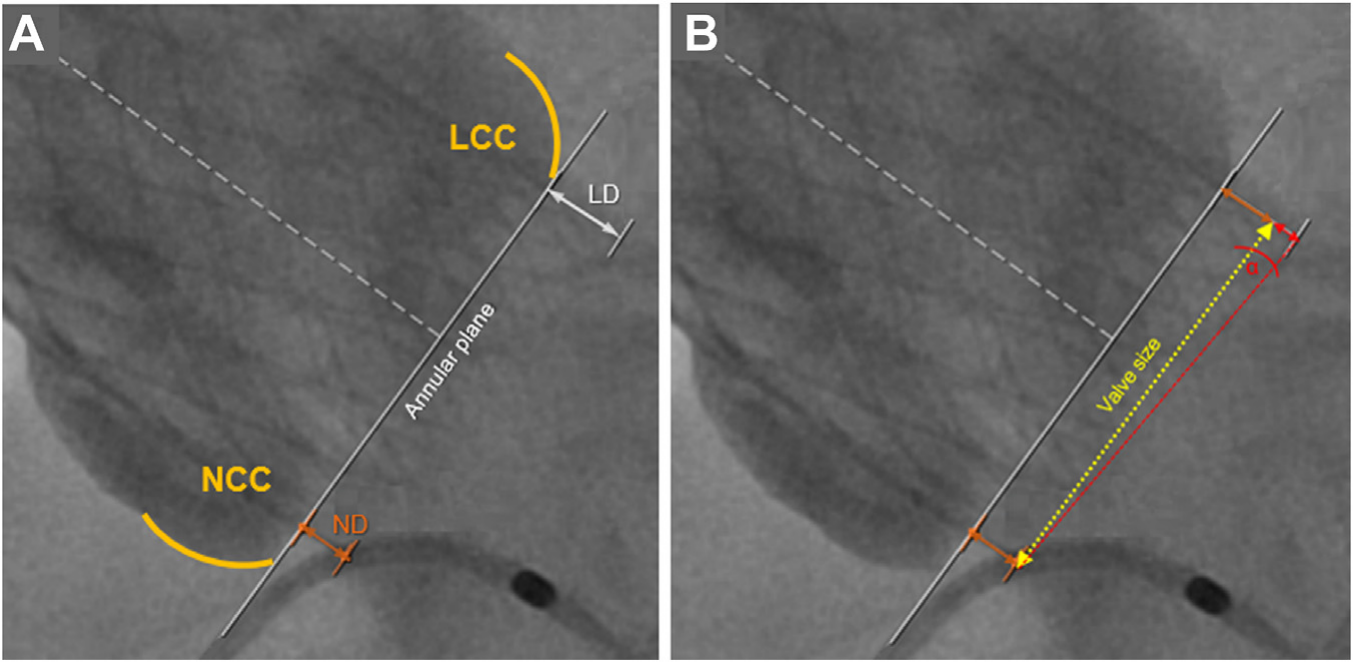
Assessment methods for implantation depth evaluation after valve deployment. **A:** Implantation depth was assessed using 4 different methods: noncoronary cusp (NCC) distance (ND), left coronary cusp (LCC) distance (LD), arithmetic mean (AM), and the deepest edge (DE) (eg, ND = 2.7 mm, LD = 4.7 mm, AM = 3.7 mm, DE = 4.,7 mm). **B:** Angle of implantation (α) was measured as arctan (LD-NDValve size)∗180π.

Evaluation of valve implantation depth was based on offline analysis of postdeployment aortic angiograms of the orthogonal view of the valve (3-cusp view). The PACS system workstation (SECTRA IDS7, Sectra, Linköping, Sweden) was used for measurement. The aortic annulus was identified by tracing a line through the nadirs of the sinuses of Valsalva (hinge points) between the noncoronary cusp (NCC) and the left coronary cusp (LCC). From this line, the NCC distance (and the LCC distance were measured as the distance from the cusp nadir to the respective end of the ventricular prosthesis (Figure 1A). The arithmetic mean of these distances and the measure of the deepest edge, regardless of the cusp side, also were taken into account. The angle of implantation in relation to the annulus plane, also known as valve tilt, was assessed as previously described (Figure 1B)^9^:

$$Valve\;tilt=\arctan\left(\frac{deepest\;edge\;-\;minor\;edge}{valve\;size}\right)*\frac{180}{\pi \;}\;\left(1\right)$$

### Calcium quantification

**Figure 2:**
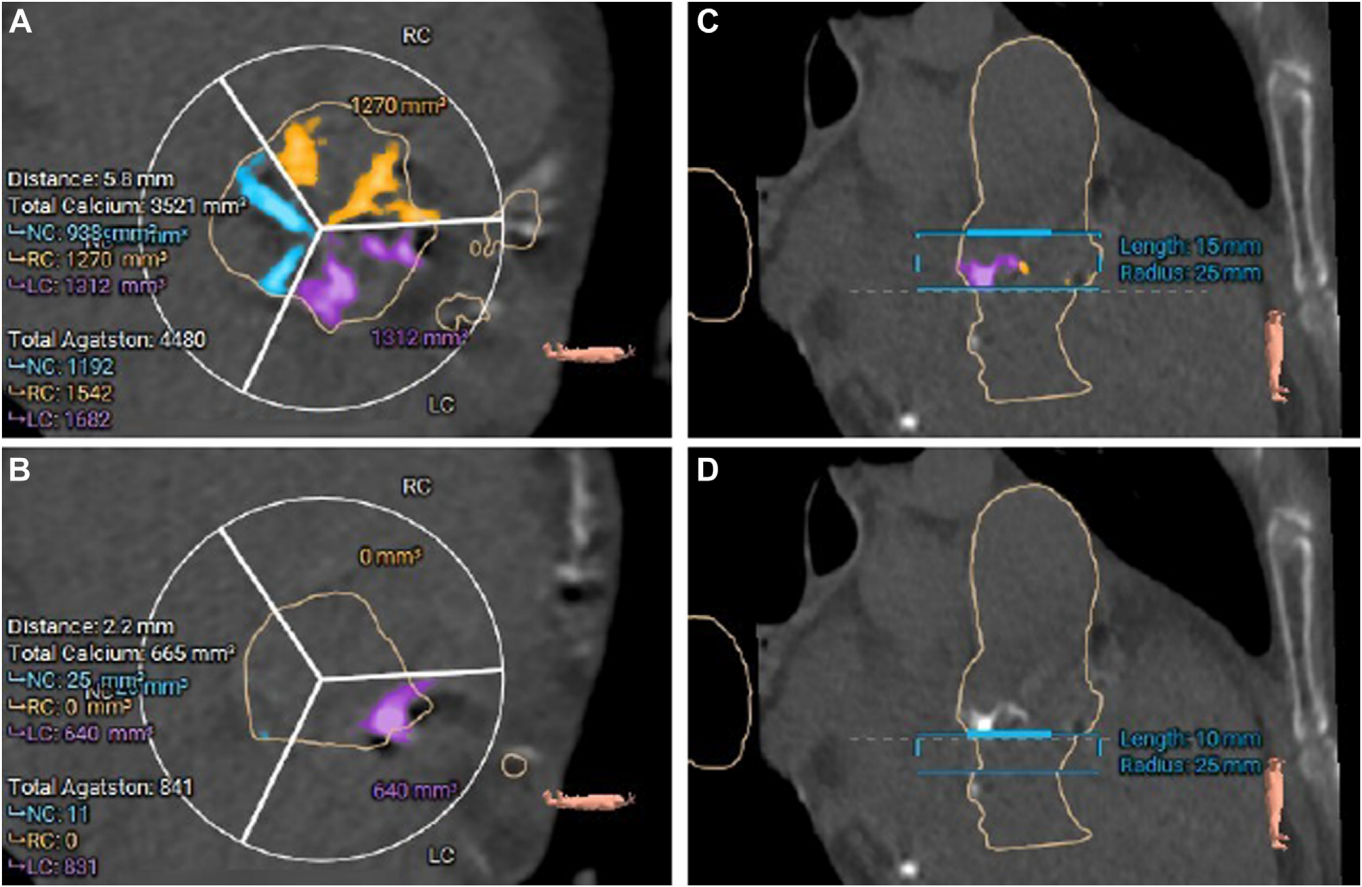
Calcium quantification of the device landing zone according to leaflet sector. **A:** Calcium load at the aortic valve complex (AVC) level: noncoronary cusp (NC) 938 mm^3^, right coronary cusp (RC) 1270 mm^3^, left coronary cusp (LC) 1312 mm^3^. **B:** Calcium at the left ventricular outflow tract (LVOT) level (LVOT_NCC_ 25 mm^3^, LVOT_RCC_ 0 mm^3^, LVOT_LCC_ 640 mm^3^). **C:** AVC was defined as the volume 15 mm above the annular plane to the lower coronary ostium. **D:** LVOT was defined as the volume 10 mm below the annular plane. LVOT_NCC_ calcification was associated with prolonged HV interval.

The pattern of calcium distribution was assessed using 3mensio® Medical Imaging (Bilthoven, The Netherlands) for all available noncontrast scans with a threshold for calcium detection set at 130 HU. We adopted the calcium volumetric methodology according to Callister et al^10^ because it showed better reproducibility than the traditional Agatston score. The region of interest for calcium quantification was defined as the volume extending 15 mm above the annular plane for the aortic valve cusps, and 10 mm below the annular plane for the left ventricular outflow tract (LVOT). To assess separately the calcification of each leaflet, the 3mensio sectorial tool was adapted to the NCC, right coronary cusp (RCC), and LCC. The corresponding regions underlying each cusp divided the LVOT calcification into LVOT_NCC_, LVOT_RCC_, and LVOT_LCC_ (Figure 2).

### Statistical analysis

Differences in continuous variables were tested with the paired and unpaired versions of the *t* test or Mann-Whitney Wilcoxon test, depending on the distribution of the variables, as appropriate. Categorical variables were evaluated using the χ^2^ test, Fisher test, or McNemar test, as appropriate. Correlation between continuous variables was tested using the Pearson or Spearman correlation coefficient, as appropriate. Twenty patients were randomly selected for the evaluation of inter-rater and intra-rater reliability. Two blinded physicians measured the depth of implantation and the calcium volume. The interclass correlation coefficient (ICC) (Pearson correlation with 2-way random/absolute agreement model) was used to assess reliability, with a value >0.8 suggesting excellent agreement and a value <0.4 indicating poor agreement. Quantile regression models (for skewed outcome variables), linear regression models (for normally distributed outcomes), and logistic regression models (for categorical outcomes) were used for predictive analysis. Multivariable regression models were evaluated using the Akaike information criterion and R^2^. The added value of a parameter was estimated using the likelihood ratio test. To minimize the risk of bias and for more accurate effect estimates, missing at random variables were statistically imputed in accordance using the “Hmisc”, “rms”, and “MICE” packages.^11^ All results were consistent in a dataset with complete cases. All statistical analysis was performed using R Version 4.1.2 (R Foundation for Statistical Computing, Vienna, Austria). *P* <.05 was considered significant.

## Results

### Baseline characteristics

A total of 138 patients underwent TAVR between June 2020 and June 2021 at our institution. Of these patients, 27 (19%) were excluded according to predefined criteria (7 with previous PMI, 6 with valve-in-valve TAVR, 3 periprocedural deaths, 2 aborted TAVR procedures, and 9 missing EP study). In 10 patients, periprocedural PMI was performed because of a complete AV block.

One hundred one patients (mean age 81 ± 5.7 years; 45% women) were included for the final analysis. In 21 cases, calcium quantification was missing because of unavailable native CT scans. Table 1 lists the baseline characteristics of the patients stratified by duration of the HV interval intraprocedure post-TAVR (HV interval >55 ms). No differences were observed between the 2 groups with regard to age and comorbidities but were noted with regard to left ventricular ejection fraction (median 55% vs 60%; *P* = .004).

**Table 1:** Baseline characteristics

Parameter	Overall	Normal HV (≤55 ms) post-TAVR∗ (N = 64)	Prolonged HV (>55ms) post-TAVR∗ (N = 37)	*P* value
Age (y)	81.3 ± 5.74	81.5 ± 5.43	80.9 ± 6.29	.534
Female	45 (44.6)	32 (50.0)	13 (35.1)	.215
Hypertension	83 (82.2)	51 (79.7)	32 (86.5)	.779
CAD	48 (47.5)	30 (46.9)	18 (48.6)	1
Diabetes mellitus	26 (25.7)	16 (25.0)	10 (27.0)	1
Dyslipidemia	49 (48.5)	33 (51.6)	16 (43.2)	.36
Previous MI	14 (13.9)	8 (12.5)	6 (16.2)	.898
Previous stroke	6 (5.9)	5 (7.8)	1 (2.7)	.404
Atrial fibrillation	33 (32.7)	20 (31.3)	13 (35.1)	.986
CKD	42 (41.6)	27 (42.2)	15 (40.5)	.096
LVEF (%)	60 [55–65]	60 [57–65]	55 [50–60]	.004

Values are given as mean ± SD, n (%), or median [interquartile range] unless otherwise indicated.

CAD = coronary artery disease; CKD = chronic kidney disease; LVEF = left ventricular ejection fraction; MI = myocardial infarction; TAVR = transcatheter aortic valve replacement.

∗ Post-TAVR indicates intraprocedure immediately after valve deployment.

### Procedural characteristics and EP measurements

At baseline, median [interquartile range] HV interval was 46 [39–50] ms. Ten patients showed pre-existing infranodal conduction delay, and 7 patients demonstrated pre-existing LBBB. Post-valve deployment, median HV interval was 52 [44–58] ms, with median delta HV of 4 [0–10] ms, and new infranodal conduction delay occurred in 27 patients (30%). Post-valve deployment, new-onset LBBB was observed in 53 patients (58%). However, 1 day after TAVR, new LBBB persisted in only 18 patients (20%). Tables 2 and 3 and Supplemental Table S1 summarize further procedural characteristics and EP measurements.

**Table 2:** Procedural characteristics stratified according to HV interval

Parameter	Overall (N = 101)	Normal HV (≤55 ms) post-TAVR∗ (N = 64)	Prolonged HV (>55 ms) post-TAVR∗ (N = 37)	*P* value
Implantation assessment				
NCC distance (mm)	5.0 ± 3.1	5.1 ± 3.3	4.9 ± 2.9	.797
LCC distance (mm)	5.7 ± 2.4	5.6 ± 2.4	5.9 ± 2.3	.392
Deepest edge (mm)	6.3 ± 2.6	6.2 ± 2.8	6.4 ± 2.4	.719
Arithmetic mean (mm)	5.4 ± 2.6	5.3 ± 2.7	5.4 ± 2.4	.888
Angle of implantation (°) (%)	2.1 (5.2)	1.9 (5.4)	2.4 (4.9)	.644
Calcium volume				
Total AVC (mm^3^)	2080 [1530–2710]	2050 [1360–2510]	2260 [1880–3020]	.129
Total LVOT (mm^3^)	207 [67.0–385]	138 [52.5–323]	283 [110–670]	.042
LCC (mm^3^)	516 [345–816]	470 [288–722]	551 [409–1090]	.171
RCC (mm^3^)	622 [375–831]	588 [375–785]	775 [462–997]	.097
LVOT_NCC_ (mm^3^)	42.0 [7.00–152]	31.0 [4.50–87.0]	104 [24.5–307]	.011
LVOT_LCC_ (mm^3^)	76.0 [22.0–156]	71.0 [22.5–147]	97.5 [19.8–209]	.435
LVOT_RCC_ (mm^3^)	11.0 [0–43.5]	8.00 [0–33.5]	23.5 [1.50–67.8]	.114
Type of valve				
Acurate	46 (45.5)	35 (54.7)	11 (29.7)	.026
Sapien	14 (13.9)	8 (12.5)	6 (16.2)	.824
Evolut	39 (38.6)	20 (31.3)	19 (51.4)	.074
Lotus Edge	2 (2)	1 (1.6)	1 (2.7)	.826

Values are given as mean ± SD, median [interquartile range], or n (%) unless otherwise indicated.

AVC = aortic valve complex; LCC = left coronary cusp; LVOT = left ventricular outflow tract; LVOT_LCC_ = left ventricular outflow tract volume below the left coronary cusp; LVOT_NCC_ = left ventricular outflow tract volume below the noncoronary cusp; LVOT_RCC_ = left ventricular outflow tract volume below the right coronary cusp; NCC = noncoronary cusp; RCC = right coronary cusp; TAVR = transcatheter aortic valve replacement.

∗ Post-TAVR indicates intraprocedure immediately after valve deployment.

**Table 3:** Electrocardiographic and electrophysiological findings

	Intraprocedural pre-valve deployment	Intraprocedural post-valve deployment	At day 1 after TAVR	*P* value pre- vs post- TAVR
LBBB	7 (8)	60 (59.4)	23 (22.8)	<.001∗
PR interval (ms)	198 ± 42.6	216 ± 44.2	189 ± 33.3	<.001†
QRS duration (ms)	110 ± 21.9	139 ± 28.5	116 ± 26.7	<.001†
AH interval (ms)	112 [94–131]	119 [96–138]	108 [84–134]	.002‡
HV interval (ms)	46 [39–50]	52 [44–58]	50 [44–53]	<.001‡
>55 ms	10 (9.9)	37 (36.6)	5 (21)	<.001∗
>70 ms	2 (2)	8 (7.9)	3 (12.5)	.08∗

Values are given as n (%), mean ± SD, or median [interquartile range] unless otherwise indicated.

LBBB = left bundle branch block; TAVR = transcatheter aortic valve replacement.

∗ McNemar’s test;

† paired *t* test;

‡ paired Wilcox test.

### Valve implantation depth and infranodal conduction delay

**Figure 3:**
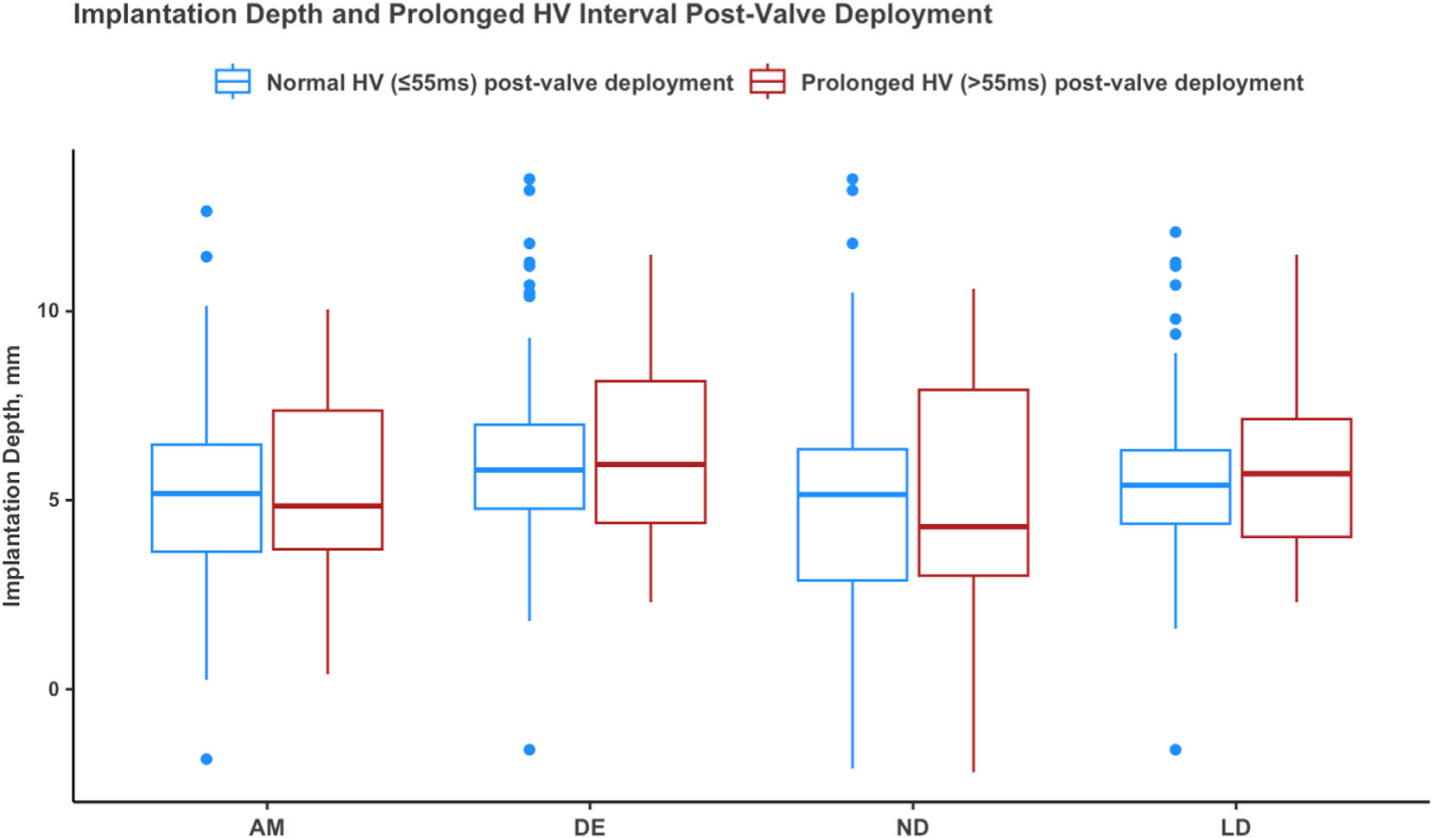
Prolonged HV interval intraprocedure post-transcatheter aortic valve replacement was not associated with the depth of valve prosthesis regardless of the method applied to determine the implantation depth. AM = arithmetic mean; DE = deepest edge; LD = left coronary cusp distance; ND = noncoronary cusp distance.

Mean implantation depth was 5. 4 ± 2.6 mm when considering the arithmetic mean, whereas mean implantation depth considering the deepest edge was 6.3 ± 2.6 mm. Implantation depth was similar between patients with normal and prolonged HV intervals post-valve deployment (NCC distance = 5 ± 3.3 mm vs 4.9 ± 2.9 mm, *P* = .79; LD= 5.6 ± 2.4 mm vs 5.9 ± 2.3 mm, *P* = .39) (Table 2). We found no correlation between delta HV and implantation depth (r = –0.08). In none of the univariable or multivariable quantile regression models did valve implantation depth predict the HV interval post-valve deployment (Figure 3). Similarly, no significant correlation was found between valve tilt and HV interval.

### Calcification and infranodal conduction delay

**Figure 4:**
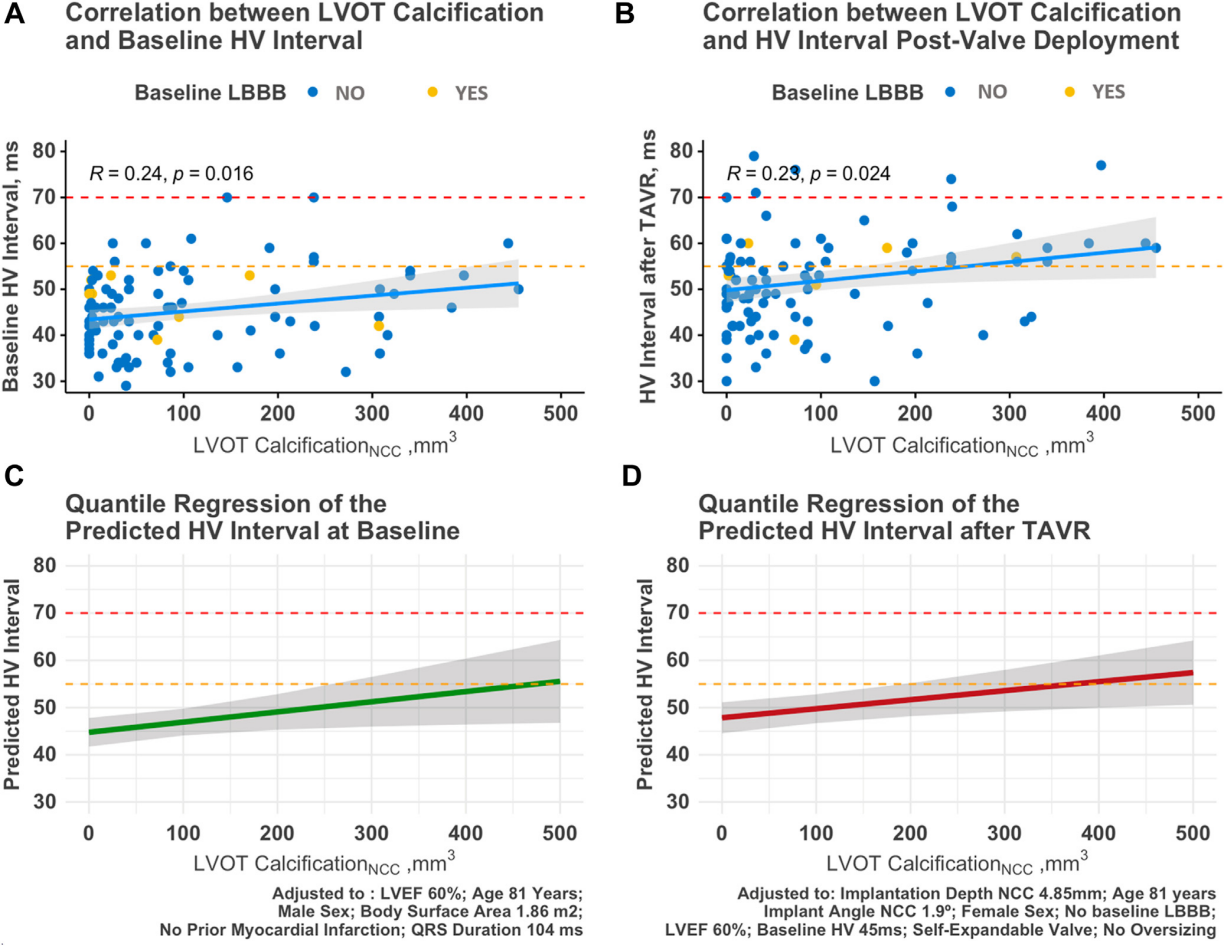
**A:** Scatterplot and Pearson correlation coefficient between left ventricular outflow tract (LVOT) calcification and baseline HV interval. **B:** Scatterplot and Pearson correlation coefficient between LVOT calcification and HV interval intraprocedural post-TAVR. **C:** Quantile regression model showing the prediction of baseline HV interval using LVOT calcification when given a series of baseline parameters (subscript). **D:** Quantile regression model showing prediction of HV interval intraprocedural post-TAVR using LVOT calcification given a series of baseline and procedural parameters (see Results). *Orange dotted line* marks the limit of 55 ms for an abnormal HV interval. *Red dotted line* marks the 70-ms cutoffs for the HV interval to trigger the pacemaker implant. “After TAVR” indicates intraprocedure immediately after valve deployment. LBBB = left bundle branch block; LVEF = left ventricular ejection fraction; NCC = noncoronary cusp; TAVR = transcatheter aortic valve replacement.

Overall, median total calcium volume at the aortic valve level was 2080 [1530–2710] mm^3^. Median total calcium volume at the LVOT level was 207 [1530–2710] mm^3^. Although there was no correlation between delta HV and total calcium volume (r = 0.12), patients with a higher burden of LVOT calcification showed a prolonged HV interval (>55 ms) post-valve deployment (*P* = .042). When LVOT calcification was stratified by the cusp sectors, the calcium volume of LVOT_NCC_ was significantly higher in the group with infranodal delay than in patients with normal HV interval (104 [24.5–307] mm^3^ vs 31 [4.5–87] mm^3^; *P* = .01) (Table 2). A weak correlation was found between LVOT_NCC_ calcium and HV interval post-valve deployment (r = 0.23) (Figure 4), whereas no correlation was found between other LVOT sectors and HV interval.

In univariable analysis using quantile regression, LVOT_NCC_ calcification was associated with the median HV post-valve deployment (β = 2.48, 95% confidence interval [CI] 0.91–4.01, per 100-mm^3^ increase, p_anova_ = 0.002) and remained associated after adjusting for age and sex (β = 2.77, 95% CI 1.48–3.85, per 100-mm^3^ increase, p_anova_ <0.001). In multivariable analysis, after adjusting for baseline characteristics (age, sex, LBBB, HV interval, and left ventricular ejection fraction) and procedural parameters (implantation depth, valve angle, type of valve, oversizing), the volume of LVOT_NCC_ calcium remained significantly associated with the median HV interval post-valve deployment (β = 2.16, 95% CI 0.46–3.3, per 100-mm^3^ increase, p_anova_ = 0.01) (Figure 4B and Supplemental Figure S2).

In patients with a normal baseline HV interval, LVOT_NCC_ calcification was able to predict a prolonged HV interval (>55 ms) post-valve deployment in the univariable (odd ratio [OR] 2.01, 95% CI 1.34–3.16, per 100-mm^3^ increase, *P* = .01) and multivariable logistic regression adjusting for age and sex (OR 2.37, 95% CI 1.5– 4.1, *P* <.001).

LVOT_NCC_ calcification added significant value to a logistic regression model containing known risk factors (baseline HV interval, age, sex, pre-existing LBBB, and type of TAVR valve) for a prolonged HV interval post-valve deployment (likelihood ratio χ^2^ 15.19; *P* <.001). The fraction of new information added by LVOT calcification to the model was 40% (Supplemental Figures S3 and S4).

### Type of valve and infranodal conduction delay

Patients who received the Acurate prosthesis showed a lower rate of infranodal conduction delay post-valve deployment (N = 35 vs 11; *P* = .026). For the other prosthesis types (Sapien, Evolut, Lotus), no significant differences between the groups was found (Table 2).

### Implantation depth, calcification, and PMI

In the secondary analysis of 10 patients who underwent PMI directly after TAVR, implantation depth was significantly lower than in patients who did not undergo PMI (arithmetic mean 8.2 ± 2.9 mm vs 5.4 ± 2.5 mm, respectively; *P* = .004). Distribution of calcium load at the aortic valve complex did not differ significantly between the groups with and without PM (Supplemental Tables S2 and Table S3).

#### Inter- and intraobserver reliability

The ICC for intraobserver reliability was very high: 0.95 (95% CI 0.87–0.97; *P* <.001) for implantation depth (arithmetic mean) and 0.91 (95% CI 0.78–0.96; *P* <.001) for the total calcium quantification, respectively. The ICC for interobserver reliability for implantation depth (arithmetic mean) and total calcium quantification was moderate, with ICC of 0.70 and 0.55, respectively.

## Discussion

The present study aimed to investigate the impact of implantation depth and calcium burden in the DLZ on infranodal conduction properties during and after TAVR. Our main findings were as follows. First, calcium volume at the level of the LVOT, especially at the LVOT_NCC,_ was significantly higher in the group with vs the group without infranodal delay immediately after TAVR. Second, LVOT_NCC_ calcification was a significant predictor of infranodal conduction delay intraprocedure pre- and post-TAVR. Third, calcium volume at the level of the aortic valve did not differ between the group with infranodal delay and the group of patients with normal HV interval. Fourth, although implantation depth was significantly deeper in patients undergoing periprocedural PM implantation, implantation depth did not correlate with prolonged HV interval.

Our findings corroborate and extend previous studies assessing implantation depth and calcium burden in TAVR patients. Calcium distribution is not uniform across the LVOT and aortic valve annulus. Buellesfeld et al^12^ reported a higher prevalence of calcification at the annular level than in the LVOT in patients with severe aortic stenosis (61% vs 36%). Whereas the analysis of the German TAVR registry by Staubach et al^13^ found no correlation between the degree of aortic valve calcification and the rate of PMI after the procedure, Fujita et al^6^ reported an increased risk of permanent PMI in patients with calcification of the LCC at the level of the aortic valve. Moreover, Mauri et al^14^ found that LVOT calcifications beneath the LCC and RCC were associated with a higher rate of PMI. In our cohort, LVOT_NCC_ calcification was the only significant predictor of infranodal conduction delay, most probably due to the vicinity to the His bundle.^15^

One other reason for the disparity in the reported results regarding aortic valve calcification is the heterogeneity of the methods used for quantification of the aortic valve calcification. In the studies of Fujita et al^6^ and Mauri et al,^14^ volumetric calcium quantification was based on contrast CT scans with a threshold of 500 HU, which then was manually adjusted if this was regarded as not appropriate for the capturing of calcification. Instead, according to Pawade et al,^16^ we used only native CT scans with a threshold of 130 HU in order to include all the calcification mass that might have been opacified by the contrast agent and therefore underestimated by the selection of a higher HU. This is in line with the standard threshold of 130 HU for vascular calcification used for the Agatston score.^17^

Although the association between low implantation depth and PMI rate after TAVR has been previously described,^18^ Reiter et al^19^ found no correlation between implantation depth and HV prolongation. Similarly, in our study, implantation depth did not differ between the group with prolonged HV interval and the group with normal HV interval intraprocedurally after TAVR. Given the heterogeneity of approaches used in the literature to define implantation depth, we investigated different measurements to assess implantation depth. Piayda et al^3^ showed that the method chosen influences the rate of achievement of the “optimal” implantation depth. In our study, none of the approaches could significantly differentiate the group with prolonged HV interval from the group with normal HV interval immediately after TAVR, thus highlighting the need for further investigation and consensus on how to best assess the implantation depth.

### Study limitations and strengths

First, our analysis shares all the known limitations of retrospective design, including selection bias. Second, the inability to obtain preprocedural native CT scans led to missing data on calcium burden in 21 cases. Third, assessment of implantation depth with angiograms is intrinsically limited by the parallax effect. Nevertheless, this is the method most commonly used in clinical practice. Fourth, the study was underpowered for prediction of PMI and evaluation of the cusp-specific risk or adjustment for other baseline and procedural characteristics. Fifth, the clinical consequences of HV interval prolongation >55 ms is debatable. However, we chose this cutoff because infranodal conduction delay is defined by HV ≥55 ms and based on our previous experience using this cutoff for risk stratification of patients with new LBBB after TAVR.^8^ Sixth, implantation depth and calcium burden might not be the only factors impacting conduction properties. Other factors, such as an acute inflammatory response,^20^ also might play a role in the development of infranodal conduction delay after TAVR but were not investigated in this study.

The strengths of our study are as follows: (1) Each patient received a comprehensive evaluation with at least 2 EP studies (3 in case of LBBB) and pre- and postprocedural CT scans; (2) the CT scans were evaluated by 2 independent cardiologists; and (3) our analysis provides further guidance in determining a general definition for the optimal assessment of valve implantation depth.

## Conclusion

Preprocedural assessment of LVOT_NCC_ calcium burden may identify patients at risk for conduction disorders after TAVR. Implantation depth was not associated with the incidence of LBBB or infranodal conduction delay.
