# Supplementary material for: Impact of implantation depth and calcium burden on infranodal conduction delay after transcatheter aortic valve replacement
Source: Heart Rhythm O2. 2023 Dec 18;5(2):113–21. doi: 10.1016/j.hroo.2023.12.003 (PMC10964348; doi:10.1016/j.hroo.2023.12.003)
Supplement: Supplemental Figures S1–S4 and Supplemental Tables S1–S3 [file mmc1.docx]

**ONLINE SUPPLEMENTAL APPENDIX**

**Legend**

**Figure S1** - Intraprocedural measurement of the HV interval. Red denotes the HV interval. Black denotes the QRS width. Figure A: before TAVR. Figure B: after TAVR.

**Figure S2** - Plot showing the regression estimates of the predictor variables in predicting the 10^th^, 25^th^, 50^th^, 75^th^ and 90^th^ percentiles of the HV interval intraprocedural post-TAVR in a linear multivariable regression model (horizontal red line) and in a quantile regression line (blue line). Horizontal dotted line: no effect. We observe that baseline HV interval LVOT calcification, LVEF and female sex are important predictors of HV interval intraprocedural post-TAVR, with high variability of the estimates in the quantile regression model depending on which quartile is predicted.

**Figure S3** - Leaf plot showing the diagnostic yield of LVOT calcification. Plotted is the pre-test probability of a patient to have an abnormal HV interval intraprocedural post-TAVR using a logistic regression model without LVOT calcification (baseline HV interval+ LVEF+ baseline LBBB+ balloon-expandable valve) vs the post-test probability after adding the LVOT calcification to the model. The red lines signify the 10^th^ and the 90^th^ percentiles of the pre vs post-test probabilities. The higher the distance between the lines, the more important the addition of the new parameter to the baseline model is.

**Figure S4** - Nomogram showing the estimated HV interval using a multivariable quantile regression model involving known risk factors for the HV interval. Due to low estimates, Age, Sex, Valve implantation Depth and Valve Angle have been omitted.

**Figure S1 - Intraprocedural measurement of the HV interval.**


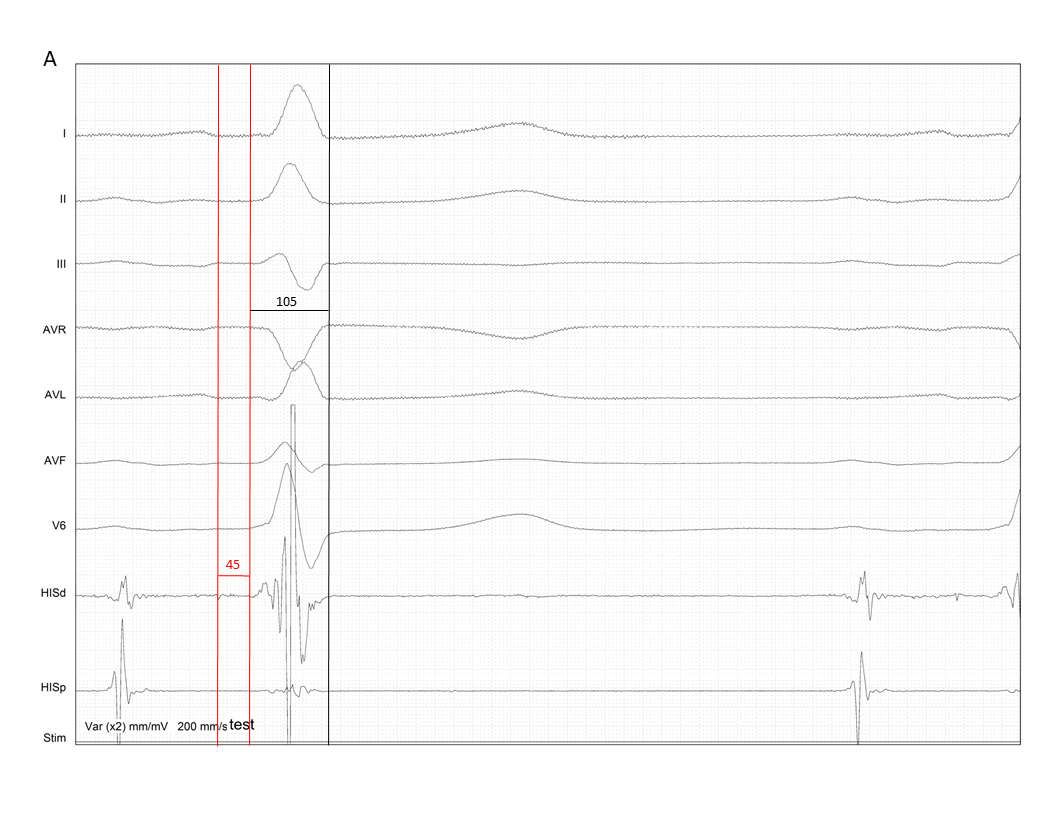

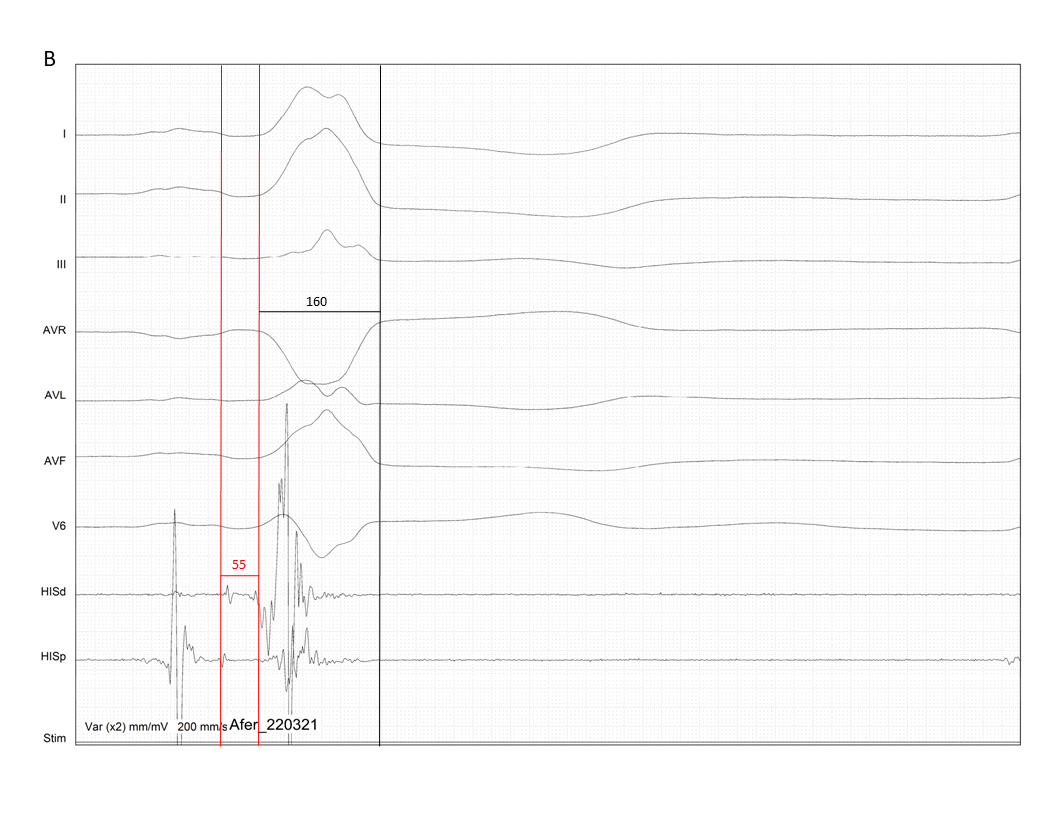


**Figure S2 - Regression estimates of the predictor variables in predicting the 10^th^, 25^th^, 50^th^, 75^th^ and 90^th^ percentiles of the HV interval after TAVR**
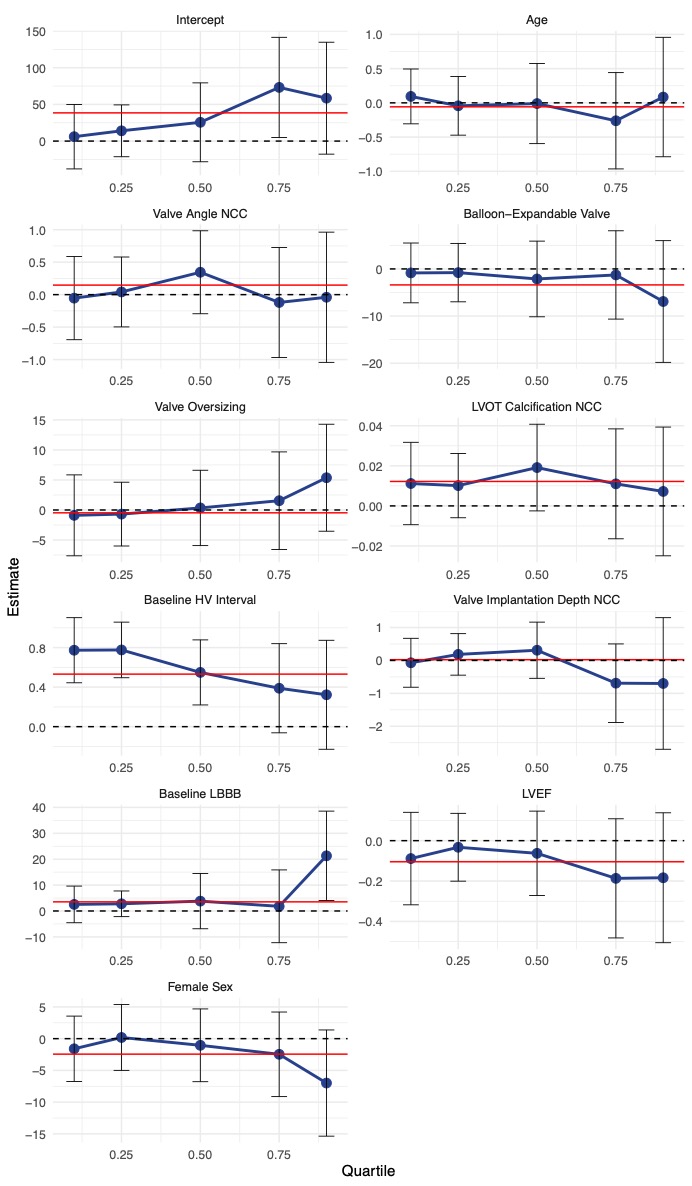


**Figure S3 - Leaf plot showing the diagnostic yield of LVOT calcification.**


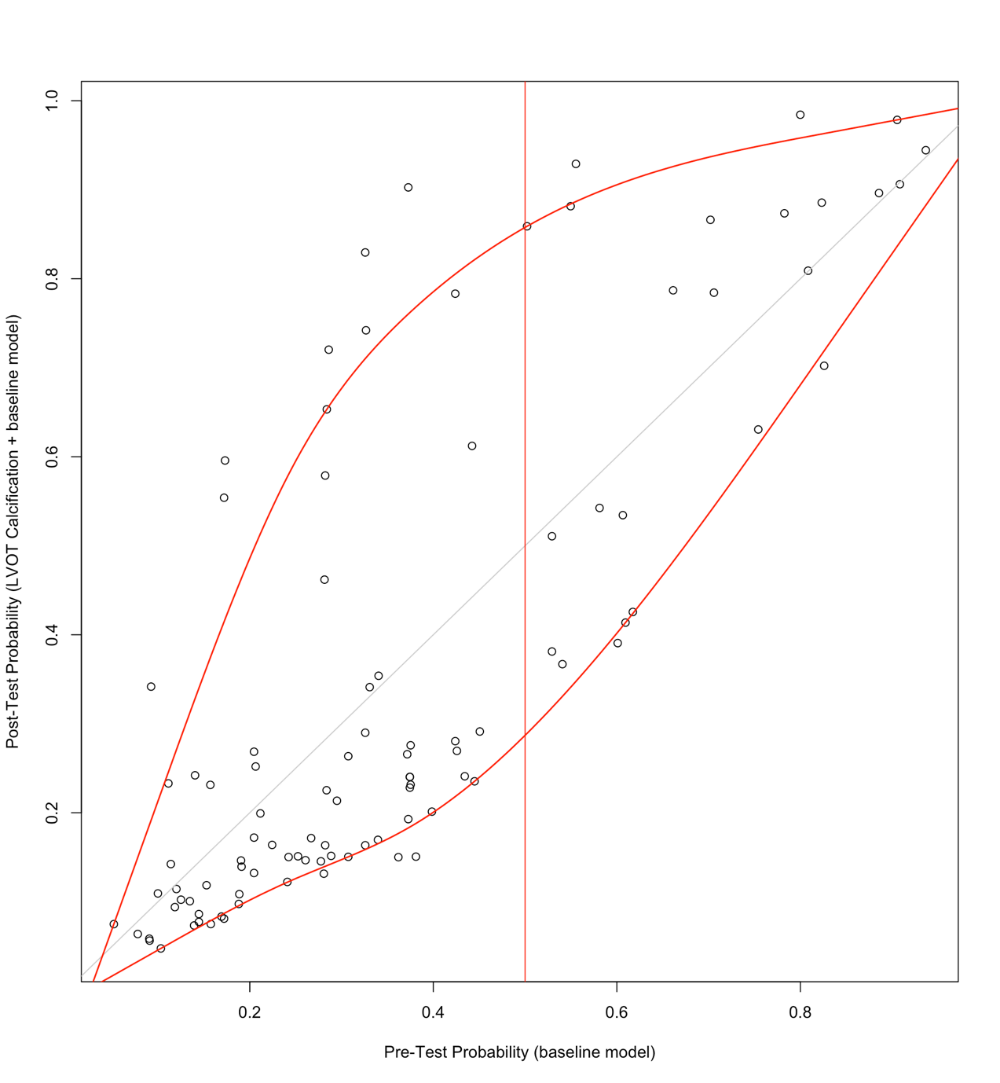


**Figure S4 - Nomogram showing the estimated HV interval using known risk factors**
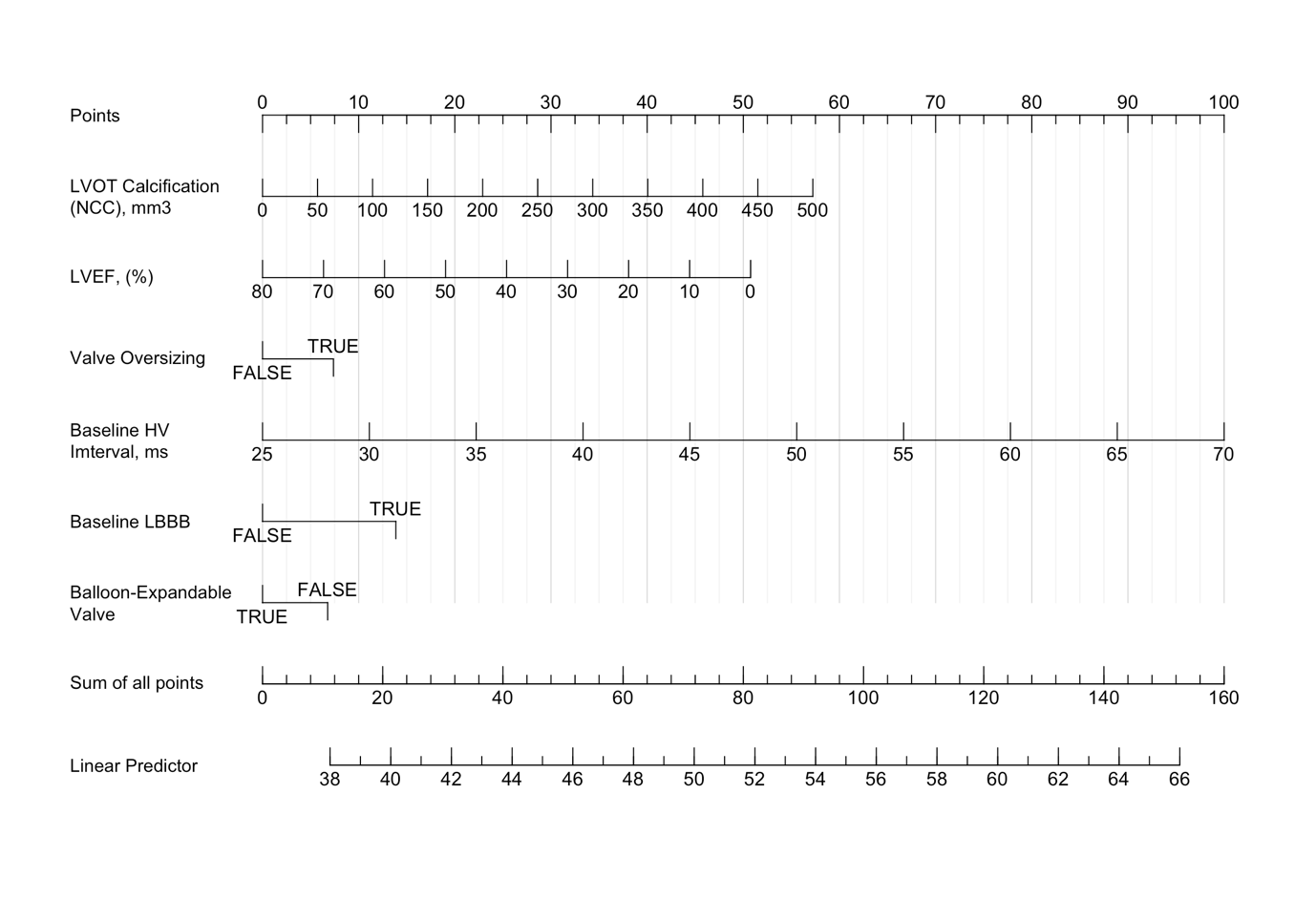


**Table S1: Procedural characteristics stratified according to new left bundle-branch-block one day after TAVR**

| **Parameter** |  | | | **Overall (N=93)** | | **No LBBB (N=74)** | | **New LBBB 1 day**  **after TAVR (N=19)** | | **P Value** |
| --- | --- | --- | --- | --- | --- | --- | --- | --- | --- | --- |
| **Implantation Assessment** | | |  |  | |  | |  | |  |
| NCC distance | mm (±SD) | | | | 4.97 (3.21) | | 4.93 (3.26) | | 5.10 (3.45) | 0.916 |
| LCC distance | mm (±SD) | | | | 5.70 (2.43) | | 5.56 (2.47) | | 6.15 (2.54) | 0.548 |
| Deepest edge | mm (±SD) | | | | 6.25 (2.71) | | 6.09 (2.74) | | 6.88 (2.85) | 0.453 |
| Arithmetic mean | mm (±SD) | | | | 5.34 (2.64) | | 5.25 (2.71) | | 5.63 (2.67) | 0.745 |
| Angle of Implantation | Degrees (%) | | | | 2.32 (5.25) | | 2.22 (4.61) | | 2.74 (7.42) | 0.62 |
| **Calcium Volume** |  | | | |  | |  | |  |  |
| Total AVC | mm^3^ [IQR] | | | | 2070 [1380, 2570] | | 2050 [1510, 2450] | | 3150 [1760, 3310] | 0.057 |
| Total LVOT | mm^3^ [IQR] | | | | 201 [67.5, 393] | | 222 [85.0, 377] | | 415 [42.5, 809] | 0.56 |
| NCC | mm^3^ [IQR] | | | | 877 [552, 1120] | | 868 [578, 1100] | | 1080 [609, 1290] | 0.29 |
| LCC | mm^3^ [IQR] | | | | 518 [339, 800] | | 505 [343, 760] | | 846 [499, 1120] | 0.117 |
| RCC | mm^3^ [IQR] | | | | 610 [369, 812] | | 596 [370, 788] | | 993 [447, 1060] | 0.061 |
| LVOT_NCC_ | mm^3^ [IQR] | | | | 42.0 [7.00, 149] | | 50.0 [9.00, 136] | | 73.0 [5.00, 335] | 0.582 |
| LVOT_LCC_ | mm^3^ [IQR] | | | | 77.5 [22.8, 162] | | 76.0 [22.0, 178] | | 141 [23.0, 251] | 0.515 |
| LVOT_RCC_ | mm^3^ [IQR] | | | | 12.0 [0, 47.8] | | 16.0 [0, 50.0] | | 4.00 [0, 135] | 0.761 |
| **Type of Valve** | | | | | | | | | | |
| Acurate Valve | | N (%) | | | 41 (44.1%) | | 33 (44.5%) | | 5 (26.3%) | 0.123 |
| Sapien Valve | | N (%) | | | 13 (14.0%) | | 9 (12.2%) | | 4 (21.1%) | 0.463 |
| Evolut Valve | | N (%) | | | 37 (39.8%) | | 28 (37.8%) | | 8 (42.1%) | 1 |
| Balloon Expandable Valve | | N (%) | | | 21 (22.6%) | | 14 (18.9%) | | 7 (36.8%) | 0.219 |

For abbreviations see Table 2

**Table S2: Calcium volume and implantation depth stratified according to new pacemaker implantation**

| **Parameter** |  | | **Overall (N=110)** | **No Pacemaker (N=101)** | **Pacemaker**  **post-TAVR* (N=10)** | **P Value** |
| --- | --- | --- | --- | --- | --- | --- |
| **Implantation Assessment** | |  |  |  |  |  |
| NCC distance | mm (±SD) | | 5.4 (3.3) | 5.0 (3.1) | 8.9 (3.4) | **0.002** |
| LCC distance | mm (±SD) | | 5.9 (2.4) | 5.7 (2.4) | 7.6 (2.4) | **0.027** |
| Deepest edge | mm (±SD) | | 6.5 (2.8) | 6.3 (2.6) | 8.9 (3.3) | **0.018** |
| Arithmetic mean | mm (±SD) | | 5.6 (2.7) | 5.4 (2.5) | 8.2 (2.9) | **0.004** |
| **Calcium Volume** |  | |  |  |  |  |
| Total AVC | mm^3^ [IQR] | | 2120 [1520, 2730] | 2080 [1530, 2710] | 2660 [1870, 2930] | 0.294 |
| Total LVOT | mm^3^ [IQR] | | 231 [66.5, 410] | 207 [67.0, 385] | 441 [147, 638] | 0.32 |
| NCC | mm^3^ [IQR] | | 890 [580, 1150] | 885 [567, 1140] | 938 [803, 1170] | 0.554 |
| LCC | mm^3^ [IQR] | | 525 [349, 926] | 516 [345, 816] | 993 [659, 1150] | 0.093 |
| RCC | mm^3^ [IQR] | | 619 [366, 841] | 622 [375, 831] | 424 [341, 894] | 0.831 |
| LVOT_NCC_ | mm^3^ [IQR] | | 42.0 [7.50, 159] | 42.0 [7.00, 152] | 38.0 [21.5, 202] | 0.63 |
| LVOT_LCC_ | mm^3^ [IQR] | | 80.0 [22.0, 173] | 76.0 [22.0, 156] | 135 [57.0, 399] | 0.289 |
| LVOT_RCC_ | mm^3^ [IQR] | | 11.5 [0, 46.3] | 11.0 [0, 43.5] | 14.0 [0, 135] | 0.754 |

For abbreviations see Table 2. *Post-TAVR is meant immediately after valve deployment

**Table S3 - Regression coefficients**

1. **Prediction of PM after valve deployment**

|  | **PM after valve deployment** | | |
| --- | --- | --- | --- |
| *Predictors* | *Odds Ratio* | *CI* | *p* |
| Intercept | 0.0078 | 0.0011 – 0.0578 | **<0.001** |
| Depth of Implantation ND, mm | 1.4226 | 1.1345 – 1.7838 | **0.003** |
| Observations | 110 | | |
| R^2^ | 0.219 | | |

1. **Prediction of a composite of PM (after TAVR and FU) and HV ≥ 70 ms (imputed dataset)**

|  | **Composite outcome** | | |
| --- | --- | --- | --- |
| *Predictors* | *Odds Ratio* | *CI* | *p* |
| Intercept | 0.1407 | 0.0530 – 0.3738 | **<0.001** |
| Depth of Implantation ND, mm | 1.0957 | 0.9496 – 1.2643 | 0.213 |
| Observations | 110 | | |
| R^2^ | 0.023 | | |

1. **Prediction of PM during follow up (excluding those with PM after valve deployment)**

|  | **PM at Follow up** | | |
| --- | --- | --- | --- |
| *Predictors* | *Odds Ratio* | *CI* | *p* |
| (Intercept) | 0.0513 | 0.0163 – 0.1272 | **<0.001** |
| HV delta | 1.0510 | 0.9927 – 1.1093 | 0.066 |
| Observations | 101 | | |
| R^2^ Tjur | 0.033 | | |

|  | **PM at Follow up** | | |
| --- | --- | --- | --- |
| *Predictors* | *Odds Ratio* | *CI* | *p* |
| Intercept | 0.1326 | 0.0380 – 0.4633 | **0.002** |
| Depth of Implantation ND, mm | 0.8977 | 0.7120 – 1.1319 | 0.364 |
| Observations | 110 | | |
| R^2^ | 0.019 | | |
